# Interaction of bleomycin, hyperthermia and a calmodulin inhibitor (trifluoperazine) in mouse tumour cells: I. In vitro cytotoxicity.

**DOI:** 10.1038/bjc.1986.14

**Published:** 1986-01

**Authors:** J. Mircheva, P. J. Smith, N. M. Bleehen

## Abstract

Evidence in the literature suggests that hyperthermia (HT) or inhibitors of calmodulin can increase the sensitivity of rodent cells to bleomycin (BLM) by interfering with DNA repair functions. In an attempt to explore methods of improving the efficacy of thermochemotherapy we have investigated the individual and combined effects of HT (44 degrees C) and the calmodulin inhibitor trifluoperazine (TFP, 30 micrograms ml-1) on early plateau phase monolayer cultures of mouse EMT6 tumour cells for simultaneous exposures to BLM. Early plateau phase cultures are relatively resistant both to HT and to BLM. The selected HT and TFP regimens (either alone or in combination) were non-toxic. Comparing the sensitizing effect (given by the ratio: Do BLM/Do BLM + modifier) of the various regimens on BLM-treated cells, we found that: TFP alone had a marginal effect (ratio 1.3), HT alone showed significant potentiation (ratio 19) and the combination of HT and TFP strongly sensitized (ratio greater than 110) cells to BLM cytotoxicity. We propose that the use of calmodulin inhibitors in thermochemotherapy is worthy of further evaluation.


*Br. J. Cancer* (1986), **53**, 99–103

### Interaction of bleomycin, hyperthermia and a calmodulin inhibitor (trifluoperazine) in mouse tumour cells: I. *In vitro* cytotoxicity

J. Mircheva[1], P.J. Smith[2] & N.M. Bleehen[2]

[1]*Department of Experimental Therapy of Tumours, Pharmacological Research Institute, Medical Academy, Sofia, Bulgaria, and* [2]*MRC Unit and University Department of Clinical Oncology and Radiotherapeutics, MRC Centre, Hills Road, Cambridge CB2 2QH, UK.*

**Summary** Evidence in the literature suggests that hyperthermia (HT) or inhibitors of calmodulin can increase the sensitivity of rodent cells to bleomycin (BLM) by interfering with DNA repair functions. In an attempt to explore methods of improving the efficacy of thermochemotherapy we have investigated the individual and combined effects of HT (44°C) and the calmodulin inhibitor trifluoperazine (TFP, $30\,\mu g\,ml^{-1}$) on early plateau phase monolayer cultures of mouse EMT6 tumour cells for simultaneous exposures to BLM. Early plateau phase cultures are relatively resistant both to HT and to BLM. The selected HT and TFP regimens (either alone or in combination) were non-toxic. Comparing the sensitizing effect (given by the ratio: $D_0$ BLM/$D_0$ BLM + modifier) of the various regimens on BLM-treated cells, we found that: TFP alone had a marginal effect (ratio 1.3), HT alone showed significant potentiation (ratio 19) and the combination of HT and TFP strongly sensitized (ratio >110) cells to BLM cytotoxicity. We propose that the use of calmodulin inhibitors in thermochemotherapy is worthy of further evaluation.

The bleomycins are a group of glycopeptide antibiotics which have activity against a variety of human and animal tumours. The clinical preparation (a copper-free mixture of bleomycins with a predominant bleomycin $A_2$ component) is used most often in combination with other chemotherapeutic agents (for review see Carter *et al.*, 1978). It is well established that bleomycin (BLM) can induce cleavage of DNA (for review see Hecht, 1979) and it is generally considered that this property is responsible for the cytotoxicity of the antibiotic.

An interesting although variable feature of *in vitro* survival curves for bleomycin-treated cells is a biphasic response in which cell killing appears to be less efficient at high drug concentrations (Terasima, 1979; for review see Twentyman, 1984). This resistance of cells does not appear to relate consistently to cells of a given cell-cycle age or subpopulations with inherent resistance (Fox, 1984), but is of obvious importance in determining the effectiveness of bleomycin in the clinical situation. It is well recognised that hyperthermia can enhance the cytotoxic effect of bleomycin (Braun & Hahn, 1975; Hahn *et al.*, 1975), and the principal benefit of such a combination treatment is the elimination of the drug-resistant portion of the survival response.

Correspondence: P.J. Smith.
Received 15 July 1985; and in revised form 14 October 1985.

It has been suggested that the mechanism for hyperthermic potentiation of BLM toxicity involves the inhibition of DNA repair functions (Meyn *et al.*, 1979). Recently it has been shown (Chafouleas *et al.*, 1984) that inhibitors of calmodulin (a calcium binding protein involved in various processes including the regulation of cell proliferation; Means *et al.*, 1982) activity can inhibit the recovery of rodent cells from potentially lethal damage induced by bleomycin, suggesting a role for calmodulin in DNA repair pathways.

We have studied the interaction of HT and a calmodulin inhibitor (trifluoperazine, TFP; Weiss *et al.*, 1982) in modifying the cytotoxic action of BLM. Our objective was to establish a non-toxic protocol for increasing the efficacy of the hyperthermia potentiation of BLM activity. We have selected an *in vitro* system which permits the generation of relatively BLM-resistant cell cultures to mimic the resistant phenotype displayed by the majority of tumour cell types *in vivo* (for review see Twentyman, 1984).

### Materials and methods

### *Cell culture*

The cells used in all experiments were EMT6/Ca/VJAC, details of which have previously been published (Twentyman & Bleehen, 1975). Cells were maintained in monolayer culture by the

© The Macmillan Press Ltd., 1986

seeding of $1.5 \times 10^5$ cells in 5 ml of Eagles MEM supplemented with 10% foetal bovine serum, 1 mM glutamine and antibiotics, and incubated in 8% $CO_2$ in air at 37°C. Culture medium was replaced with fresh medium 2 h prior to experimental manipulations.

*Drugs*

Bleomycin sulphate (Lot No. U9U10AS8; Nippon Kayaku Co., Tokyo) was donated by Lundbeck Ltd (Luton, UK) with a potency originally assayed as 1.7 mg(potency) $mg^{-1}$ solid. Manufacturer's analyses indicated that 68.7% of the preparation was bleomycin $A_2$ and copper content was <0.008%. All experiments involved freshly prepared bleomycin stock solutions in PBS A and exact concentrations of drug were determined spectrophotometrically (absorbance of a 1% solution at 294 nm, using a 1 cm path length taken as 121.5). All bleomycin (BLM) concentrations refer to weight of solid drug in complete growth medium. Trifluoperazine (TFP) was obtained from Sigma and filter-sterilised stock solutions (2000 $\mu g\,ml^{-1}$ distilled water) were prepared immediately prior to use.

*Cell survival*

Cells were detached from treated flasks by a brief exposure to trypsin/versene, the cells were counted and diluted in pre-warmed growth medium dispensed at $2.5 \times 10^2 - 2.5 \times 10^3$ cells per 9 cm plastic dish and incubated at 37°C for 10 days for the estimation of viability by clonogenic potential. Cell survival curves were generated by the analyses of collected data points using a linear transformation of the multi-target equation as described by Watson (1978).

*Hyperthermia treatment*

Flasks containing monolayer cultures were sealed using paraffin-wax film and submerged in a rapidly stirred water bath maintained at 44°C for the period specified. Temperature equilibration occured within 1 min.

### Results

*Effect of culture age on bleomycin and hyperthermia toxicity*

A previous study (Twentyman & Bleehen, 1975) had indicated that the sensitivity of EMT6 cells to bleomycin varied during the life of the monolayer cultures. We report here, preliminary studies to establish standard culture conditions for the

**Figure 1** *In vitro* growth of mouse EMT6 cells following an initial inoculum of $1.5 \times 10^5$ cells. (●) no medium change; (○) medium replaced at 1 day intervals starting on day 2.

generation of cells used in the main investigation on the interaction of HT, TFP and BLM.

Figure 1 shows the change in the number of cells per flask with time after inoculation of $1.5 \times 10^5$ cells, either with or without daily medium changes starting on day 1. Similar initial experimental growth rates were achieved under both conditions, although the re-feeding of cultures permitted a higher cell number to be achieved and prevented early cell detachment. Unfed cultures reached plateau phase between day 2 and day 4. Flow cytometric analyses (experimental data not shown) of cell cycle distributions indicated that unfed cells on day 4 comprised 74.9% $G_1$ phase, 15.2% S phase and 9.9% $G_2+M$ phase compared to 25.8% $G_1$ phase, 47.3% S phase and 26.9% $G_2+M$ phase for exponentially growing cultures. The bleomycin toxicity data shown in Figure 2 demonstrate the expected resistance of early plateau phase cultures (Twentyman & Bleehen, 1975) and the corresponding hyperthermia toxicity data presented in Figure 3 indicate that early plateau phase cultures are also more resistant to hyperthermia (44°C).

A standard cell culture protocol of unfed cultures maintained for 4 days was selected on the basis of the above experiments. The rationale being as follows: (i) the procedure results in the earliest generation of the plateau phase state without culture overcrowding, (ii) early plateau phase cultures are more resistant to heat and bleomycin and therefore offer a suitable case for the use of an agent such as TFP in the interaction studies, and (iii) it is important that in the biophysical studies (Smith *et al.*, 1985) a relatively homogenous population is analysed (75% $G_1$, for day 4 cultures), so that the results obtained for collected

**Figure 2** Effect of culture age on the bleomycin (2h drug exposure) survival responses of mouse EMT6 cells. (●) day 4 (early plateau phase) cultures; (○) day 2, (exponential phase) cultures. Datum points represent values from individual experiments.

**Figure 3** Effect of culture age on the hyperthermia responses of mouse EMT6 cells. (●) day 4 (early plateau phase) cultures; (○) day 2 (exponential phase) cultures. Datum points represent values (±s.e.) from individual experiments.

cell populations are representative of events within individual cells.

*Potentiation of BLM toxicity by HT and TFP*

A series of preliminary studies established that early plateau phase cultures of EMT6 cells were sensitized to BLM to a greater extent when both drug and heat treatments were simultaneous,

**Figure 4** Treatment protocols for mouse EMT6 cells exposed to hyperthermia (HT), TFP, BLM or a combination of agents.

**Figure 5** Effects of hyperthermia and TFP on the cytotoxicity of BLM towards early plateau phase EMT6 cells. Data derived from two independent experiments (s.e. < 15%). Refer to **Figure 4** for treatment protocols. (○) control; (●) HT; (△) TFP; (▲) combined HT and TFP treatment.

inkeeping with the findings of Lin *et al.* (1983). In attempting to design a treatment protocol in which the effects of the individual components were maximized and which would also permit interpretation at the level of biophysical studies we have employed the schedule outlined in Figure 4. The schedule allows for a 15 min pretreatment period for TFP exposures and the simultaneous treatment of cells with BLM, TFP and HT. This approach was adopted in the survival studies shown in Figure 5. The HT and TFP treatments employed were not toxic, in the absence of BLM, to early plateau phase cultures (Table I). The survival curves can be compared on the basis of the $D_0$ values (Table I). TFP alone produced a marginal potentiation of BLM toxicity the effect mainly being a reduction in extrapolation number (*n*

**Table I** BLM survival curve parameters for early plateau phase EMT6 cells

| *Treatment*[a] | *Viability*[b] (*%P.E.*) | *BLM cytotoxicity*[c] $D_0$ (*95% conf. limits*) | $D_0$ *control* / $D_0$ *treated* |
|---|---|---|---|
| control | 77±1 | 28.1 (18.5–58.7) | set at 1.0 |
| TFP | 77±9 | 21.4 (17.9–26.6) | 1.3 |
| HT | 77±8 | 1.5 (1.1–2.7) | 18.7 |
| HT+TFP | 73±2 | 0.25 (0.18–0.4) | 112.4 |

[a]See **Figure 4** for details; [b]Mean absolute plating efficiency (±s.e.) for 4 experiments; [c]Parameters derived from data in **Figure 5**.

control = 1.38; *n* TFP = 0.43). HT alone strongly enhanced BLM toxicity and the combination of HT and TFP produced a high degree of sensitization. Comparing the various treatments on the basis of the ratio [$D_0$ BLM/$D_0$ BLM + modifier] indicates a synergistic interaction between HT and TFP in enhancing BLM cytotoxicity some 112-fold.

### Discussion

In this report we provide evidence that non-toxic doses of a calmodulin inhibitor (TFP) can interact with sub-toxic levels of HT greatly to enhance the sensitivity of mouse EMT6 tumour cells to BLM. The effects were observed in early plateau phase cultures of cells which were shown to exhibit a resistant phenotype for both BLM and HT cytotoxicity. Our results suggest a role for calmodulin in controlling BLM sensitivity and perhaps the drug potentiation effects of HT.

Twentyman (1984) has noted that there is no general consensus of opinion regarding the cell-cycle phase specificity of BLM despite considerable interest in the possibility that BLM is selectively toxic to non-cycling cells. EMT6 cells are most resistant to BLM when in early plateau phase. Maintenance of cells in plateau phase for prolonged periods results in an increase in sensitivity eventually surpassing that of exponential phase cells (Twentyman & Bleehen, 1975; Twentyman, 1976). In our HT/TFP interaction studies we have selected the most resistant situation (i.e. early plateau phase) in which the majority of the cells are in a $G_1$ arrested state. Although phenothiazines, such as TFP, have pleiotropic effects on mammalian cells (Osborn & Weber, 1980; Means *et al.*, 1982) it is reasonable to assume that the BLM-sensitization effects relate to calmodulin dependent processes given the similar effectiveness of the structurally dissimilar anticalmodulin drug, W13 (Chafouleas *et al.*, 1984). It is possible that intracellular levels of calmodulin may dictate the cell-cycle phase responsiveness to BLM, since specific synthesis of this protein occurs at the $G_1$/S boundary (Means *et al.*, 1982). Thus, in early plateau phase cultures, a high level of calmodulin could provide an optimal environment for DNA repair (Chafouleas *et al.*, 1984) and hence contribute to cellular resistance. Calmodulin has a half-life of ~24 h in exponentially growing mouse 3T3 cells (Chafouleas *et al.*, 1981) suggesting that cells held for a prolonged period in a $G_1$ arrested state may become depleted of calmodulin with a concomitant reduction in cellular resistance to BLM. In any case, the critical dependence of anti-calmodulin drugs on cell-cycle age for their BLM-sensitizing effects is clearly shown by the high degree of sensitization observed in exponentially growing rodent cells (Chafouleas *et al.*, 1984) compared to the marginal level of sensitization noted in our current experiments.

It is appropriate to consider the mechanisms by which the BLM-sensitization occur. TFP is known to have significant effects on the organization of submembranous microfilaments in rodent cells resulting in changes in cell surface morphology (Osborn & Weber, 1980). Interestingly, the BLM-sensitizing agents ethanol (Mizuno, 1981) and hyperthermia (*vide supra*) apparently inactivate cells by a similar process which appears to involve changes in membrane fluidity (Li *et al.*, 1980). Furthermore, our finding of an unusual cytotoxic interaction between HT, TFP and BLM is similar to the reported synergistic interaction between ethanol and hyperthermia in inducing cell sensitization to BLM (Ishida & Mizuno, 1981). Such correlations suggest a similar mechanism for sensitization, perhaps involving membrane structures (e.g. nuclear membranes) or membrane dependent structures. The roles of DNA repair and events at the nuclear matrix in cells sensitized to the action of BLM are explored in the accompanying paper (Smith *et al.*, 1986).

We conclude that the combination of anti-calmodulin drugs in antitumour treatment regimens involving chemotherapy and hyperthermia is worthy of further evaluation *in vitro* and *in vivo*.

The authors thank Ms C.O. Anderson for technical assistance. J.M. was in receipt of a visiting Fellowship of the International Atomic Energy Agency.

### References

BRAUN, J. & HAHN, G.M. (1975). Enhanced cell killing by bleomycin and 43° hyperthermia and the inhibition of recovery from potentially lethal damage. *Cancer Res.*, **35**, 2921.

CARTER, S.E., CROOKE, S.T. & UMEZAWA, H. (1978). *Bleomycin – Current Status and New Developments*. New York, Academic Press.

CHAFOULEAS, J.G., BOLTON, W.E. & MEANS, A.R. (1984). Potentiation of bleomycin lethality by anticalmodulin drugs: a role for calmodulin in DNA repair. *Science*, **224**, 1346.

CHAFOULEAS, J.G., PARDUE, R.L., BRINKLEY, B.R., DEDMAN, J.R. & MEANS, A.R. (1981). Regulation of intracellular levels of calcodulin and tubulin in normal and transformed cells. *Proc. Natl Acad. Sci. USA*, **78**, 996.

FOX, M. (1984). Drug resistance and DNA repair. In *Anti-tumour Drug Resistance*, Fox, B.W. and Fox, M. (eds) p. 335. Springer-Verlag: Berlin.

HAHN, G.M., BRAUN, J. & HAR-KEDAR, I. (1975). Thermochemotherapy: Synergism between hyperthermia (42–43°) and adriamycin (or bleomycin) in mammalian cell inactivation. *Proc. Natl Acad. Sci. USA*, **72**, 937.

HECHT, S.M. (1979). *Bleomycin: Biochemical and Biological Aspects*. Springer-Verlag: New York.

ISHIDA, A. & MIZUNO, S. (1981). Synergistic enhancement of bleomycin cytotoxicity toward tumour cells in culture by a combination of ethanol and moderate hyperthermia. *Gann*, **72**, 455.

LI, G.C., SHIU, E.C. & HAHN, G.M. (1980). Similarities in cellular inactivation by hyperthermia or by ethanol. *Radiat. Res.*, **82**, 257.

LIN, P.-S., HEFTER, K. & JONES, M. (1983). Hyperthermia and bleomycin schedules on V79 Chinese hamster cell cytotoxicity *in vitro*. *Cancer Res.*, **43**, 4557.

MEANS, A.R., TASH, J.S. & CHAFOULEAS, J.G. (1982). Physiological implications of the presence, distribution, and regulation of calmodulin in eukaryotic cells. *Physiol. Rev.*, **62**, 1.

MEYN, R.E., CORRY, P.M., FLETCHER, S.G. & DEMETRIADES, M. (1979). Thermal enhancement of DNA strand breakage in mammalian cells treated with bleomycin. *Int. J. Radiat, Oncol. Biol. Phys.*, **5**, 1487.

MIZUNO, S. (1981). Ethanol-induced cell sensitization to bleomycin cytotoxicity and the inhibition of recovery from potentially lethal damage. *Cancer Res.*, **41**, 4111.

OSBORN, M. & WEBER, K. (1980). Damage of cellular functions by trifluoperazine, a calmodulin-specific drug. *Exp. Cell Res.*, **130**, 484.

SMITH, P.J., MIRCHEVA, J. & BLEEHEN, N.M. (1986). Interaction of bleomycin, hyperthermia and a calmodulin inhibitor (trifluoperazine) in mouse tumour cells: II DNA damage, repair and chromatin changes. *Br. J. Cancer*, **53**, 105.

TERASIMA, T., WATANABE, M. & TAKEBE, Y. (1979). Upward-concave dose-response relationship in bleomycin lethality of mammalian cells. In *Bleomycin: Chemical, Biochemical and Biological Aspects*, Hecht, S.M. (ed) p. 297. Springer-Verlag: New York.

TWENTYMAN, P.R. (1976). Comparative chemosensitivity of exponential versus plateau phase cells in both *in vitro* and *in vivo* model systems. *Cancer Chemother. Rep.*, **60**, 1719.

TWENTYMAN, P.R. (1984). Bleomycin – mode of action with particular reference to the cell cycle. *Pharmac. Ther.*, **23**, 417.

TWENTYMAN, P.R. & BLEEHEN, N.M. (1975). Changes in the sensitivity to cytotoxic agents occurring during the life history of monolayer cultures of a mouse tumour cell line. *Br. J. Cancer*, **31**, 68.

WATSON, J.V. (1978). A linear transform of the multi-target survival curve. *Br. J. Radiol.*, **51**, 534.

WEISS, B., PROZIALECK, W.C. & WALLACE, T.L. (1982). Interaction of drugs with calmodulin. Biochemical, pharmacological and clinical implications. *Biochem. Pharmacol.*, **31**, 2217.


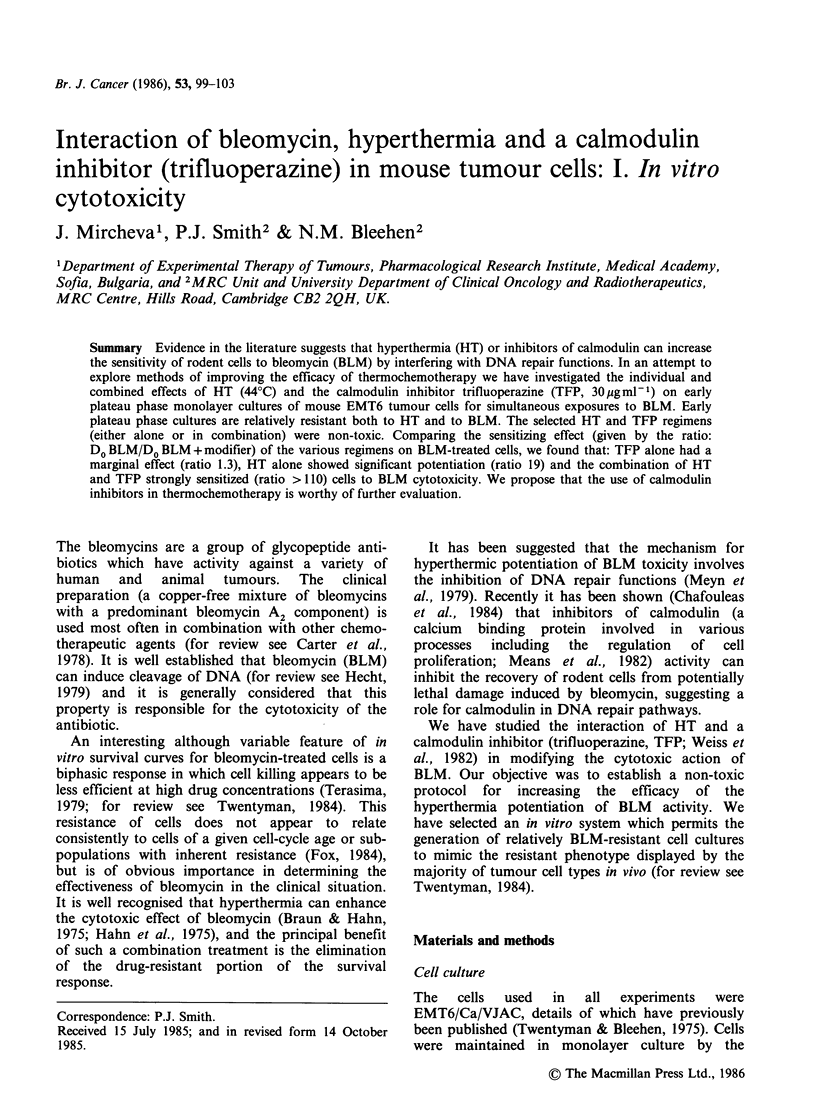

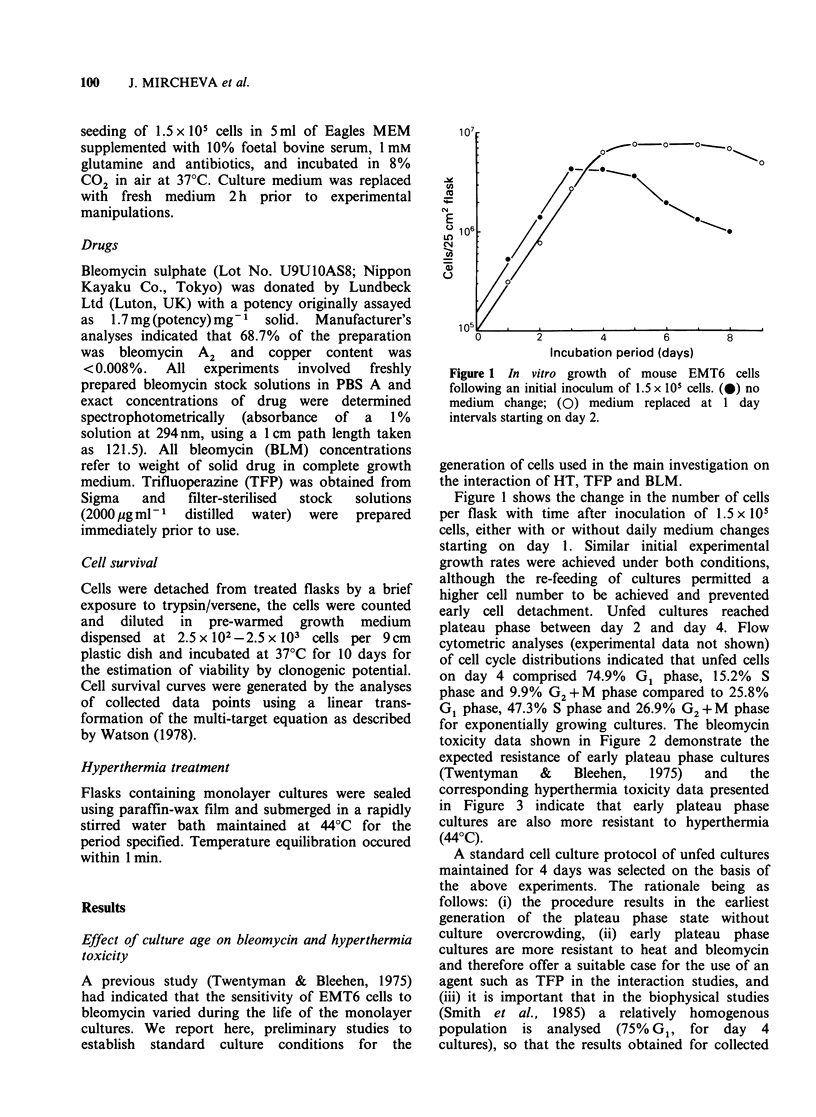

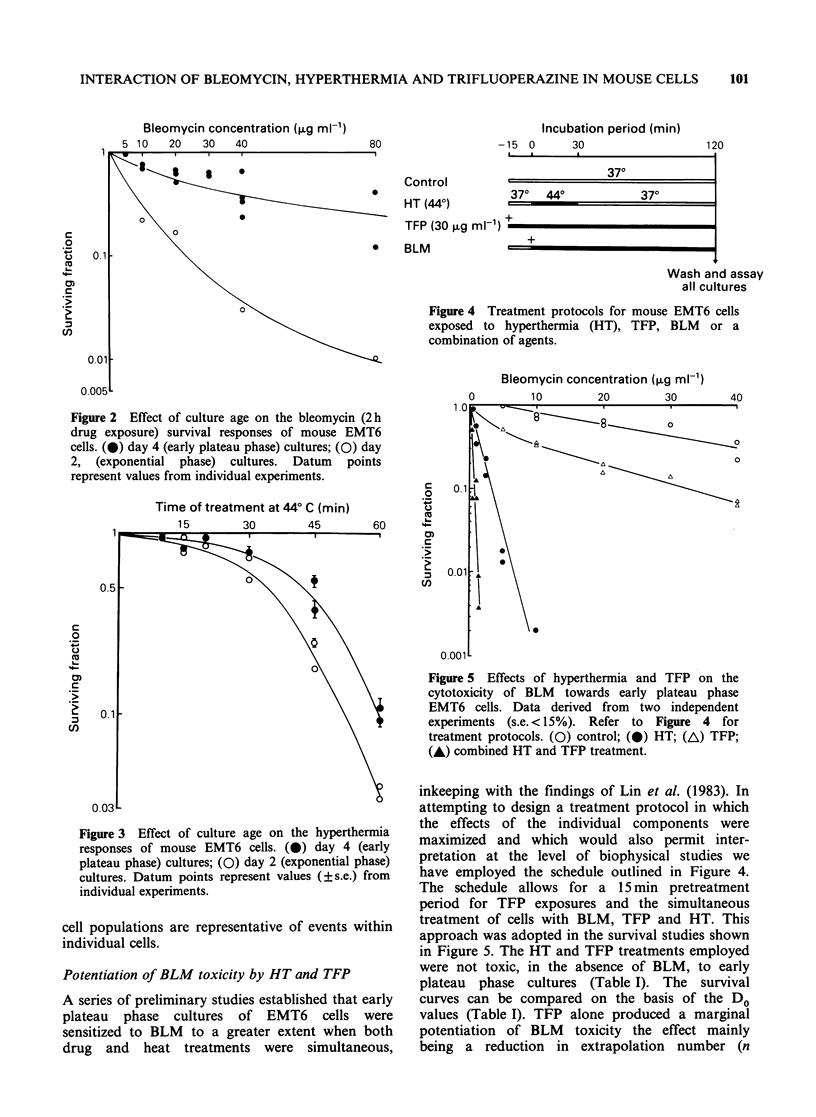

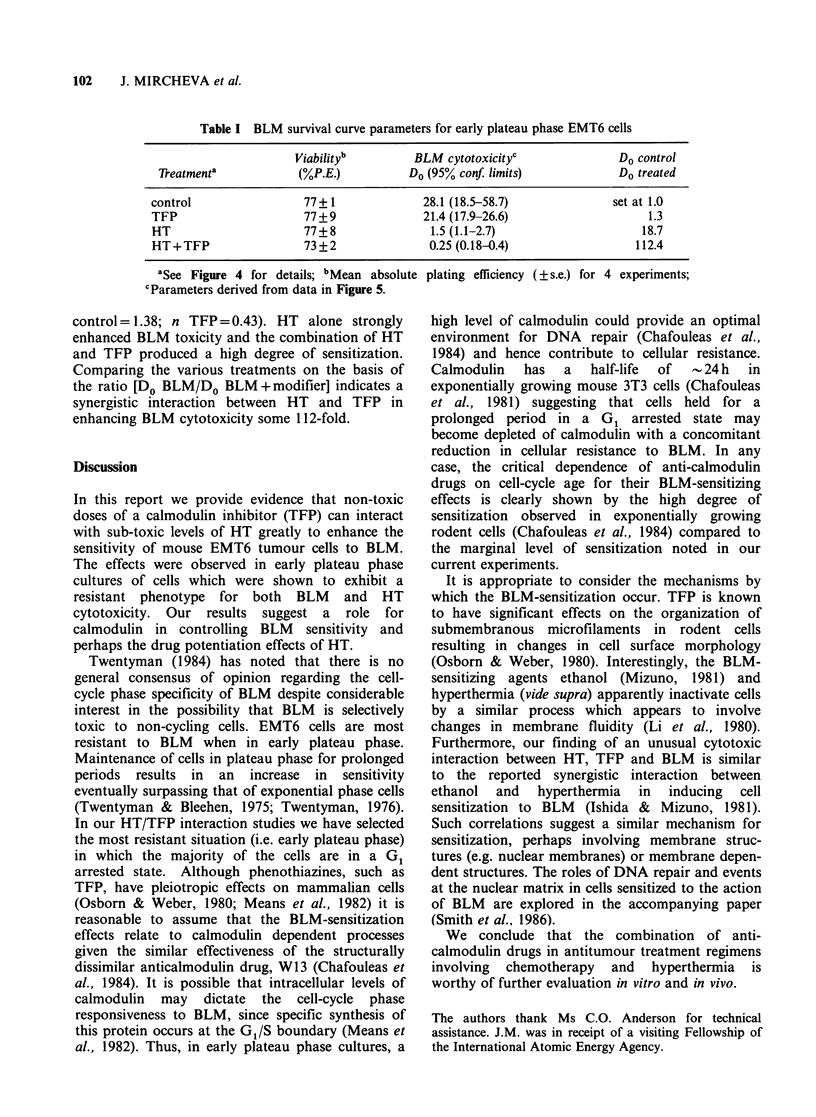

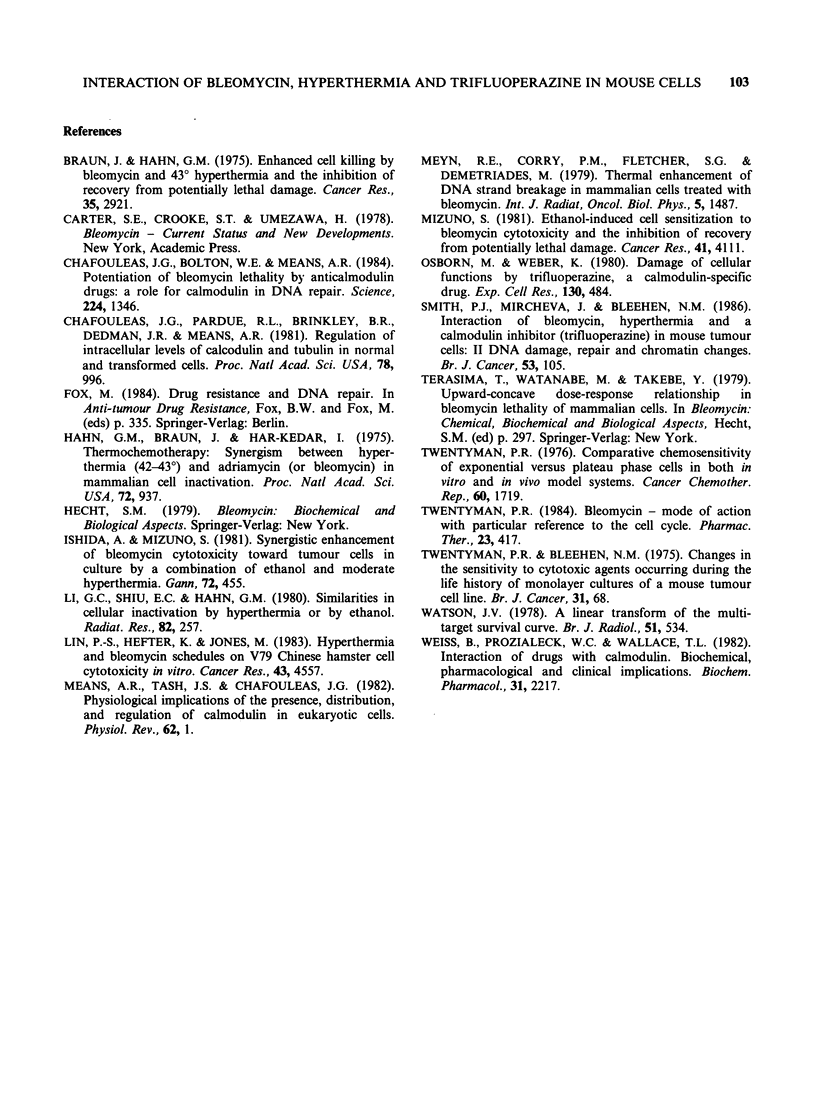


## References

[OCR_00448] Braun J., Hahn G. M. (1975). Enhanced cell killing by bleomycin and 43 degrees hyperthermia and the inhibition of recovery from potentially lethal damage.. Cancer Res.

[OCR_00459] Chafouleas J. G., Bolton W. E., Means A. R. (1984). Potentiation of bleomycin lethality by anticalmodulin drugs: a role for calmodulin in DNA repair.. Science.

[OCR_00465] Chafouleas J. G., Pardue R. L., Brinkley B. R., Dedman J. R., Means A. R. (1981). Regulation of intracellular levels of calmodulin and tubulin in normal and transformed cells.. Proc Natl Acad Sci U S A.

[OCR_00477] Hahn G. M., Braun J., Har-Kedar I. (1975). Thermochemotherapy: synergism between hyperthermia (42-43 degrees) and adriamycin (of bleomycin) in mammalian cell inactivation.. Proc Natl Acad Sci U S A.

[OCR_00488] Ishida A., Mizuno S. (1981). Synergistic enhancement of bleomycin cytotoxicity toward tumor cells in culture by a combination of ethanol and moderate hyperthermia.. Gan.

[OCR_00494] Li G. C., Shiu E. C., Hahn G. M. (1980). Similarities in cellular inactivation by hyperthermia or by ethanol.. Radiat Res.

[OCR_00499] Lin P. S., Hefter K., Jones M. (1983). Hyperthermia and bleomycin schedules on V79 Chinese hamster cell cytotoxicity in vitro.. Cancer Res.

[OCR_00504] Means A. R., Tash J. S., Chafouleas J. G. (1982). Physiological implications of the presence, distribution, and regulation of calmodulin in eukaryotic cells.. Physiol Rev.

[OCR_00510] Meyn R. E., Corry P. M., Fletcher S. E., Demetriades M. (1979). Thermal enhancement of DNA strand breakage in mammalian cells treated with bleomycin.. Int J Radiat Oncol Biol Phys.

[OCR_00516] Mizuno S. (1981). Ethanol-induced cell sensitization to bleomycin cytotoxicity and the inhibition of recovery from potentially lethal damage.. Cancer Res.

[OCR_00521] Osborn M., Weber K. (1980). Damage of cellular functions by trifluoperazine, a calmodulin-specific drug.. Exp Cell Res.

[OCR_00526] Smith P. J., Mircheva J., Bleehen N. M. (1986). Interaction of bleomycin, hyperthermia and a calmodulin inhibitor (trifluoperazine) in mouse tumour cells: II. DNA damage, repair and chromatin changes.. Br J Cancer.

[OCR_00546] Twentyman P. R. (1983). Bleomycin--mode of action with particular reference to the cell cycle.. Pharmacol Ther.

[OCR_00540] Twentyman P. R. (1976). Comparative chemosensitivity of exponential- versus plateau-phase cells in both in vitro model systems.. Cancer Treat Rep.

[OCR_00557] Watson J. V. (1978). A linear transform of the multi-target survival curve.. Br J Radiol.

[OCR_00561] Weiss B., Prozialeck W. C., Wallace T. L. (1982). Interaction of drugs with calmodulin. Biochemical, pharmacological and clinical implications.. Biochem Pharmacol.

